# Giant Retroperitoneal Lumbar Schwannoma with Extensive Vertebral Body Erosion Managed Without Spinal Instrumentation: The Potential Role of Hounsfield Unit Assessment in Surgical Decision-Making

**DOI:** 10.3390/jcm15124462

**Published:** 2026-06-09

**Authors:** Leonardo Anselmi, Luca Raspagliesi, Agostino Petroselli, Donato Creatura, Pietro Paolo Cotrufo, Emanuele Stucchi, Mario De Robertis, Ali Baram, Gabriele Capo, Laura Samà, Laura Ruspi, Maurizio Fornari, Federico Pessina, Ferdinando Carlo Maria Cananzi, Carlo Brembilla

**Affiliations:** 1 Department of Neurosurgery, IRCCS Humanitas Research Hospital, Via Manzoni 56, 20089 Milan, Italy; leonardo.anselmi@humanitas.it (L.A.); donato.creatura@humanitas.it (D.C.); mario.derobertis@humanitas.it (M.D.R.); ali.baram@humanitas.it (A.B.); gabriele.capo@humanitas.it (G.C.); maurizio.fornari@humanitas.it (M.F.); carlo.brembilla@humanitas.it (C.B.); 2 Department of Biomedical Sciences, Humanitas University, Via Rita Levi Montalcini 4, 20072 Milan, Italy; agostino.petroselli@humanitas.it (A.P.); pietro.cotrufo@humanitas.it (P.P.C.); emanuele.stucchi@humanitas.it (E.S.); laura.sama@humanitas.it (L.S.); laura.ruspi@humanitas.it (L.R.); ferdinando.cananzi@hunimed.eu (F.C.M.C.); 3 Sarcoma, Melanoma and Rare Tumor Unit, IRCCS Humanitas Research Hospital, Via Manzoni 56, 20089 Milan, Italy

**Keywords:** giant spinal schwannoma, vertebral body erosion, bone remodeling, surgical decision-making

## Abstract

**Background**: Giant retroperitoneal schwannomas with vertebral body erosion are exceedingly rare, and the decision regarding spinal instrumentation following tumor resection remains controversial in the absence of established guidelines. A 25% vertebral body involvement threshold has been proposed as an indication for fixation, yet this criterion does not account for bone quality or the potential biological adaptation of bone to chronic mechanical loading. **Case Presentation**: A 56-year-old man presented with bilateral gluteal pain and urinary urgency secondary to a giant retroperitoneal lumbar schwannoma (97 × 67 mm) with 36.6% erosion of the L5 vertebral body, confirmed by CT-guided biopsy (S100+, SOX10+, Ki-67 < 5%). Despite erosion exceeding the proposed instrumentation threshold, complete tumor resection was performed via an anterior laparotomic approach without spinal fixation, based on the absence of clinical or radiological signs of instability and the integrity of the intervertebral disc and posterior ligamentous complex. Intraoperative neurophysiological monitoring guided sacrifice of the tumor-origin root. The postoperative course was uneventful, with complete resolution of symptoms and no new complaints or neurological deficits at one-year follow-up. **Conclusions**: Post-hoc Hounsfield Unit measurements on pre-operative CT demonstrated markedly elevated bone density at the eroded L5 vertebral body (480 HU) compared with the adjacent L4 vertebra (317 HU), consistent with compensatory sclerosis induced by chronic mechanical compression. Pre-operative HU assessment may represent a valuable, readily available adjunct to anatomical erosion criteria in the surgical decision-making process for giant schwannomas with vertebral body involvement.

## 1. Introduction

Schwannomas are benign tumors arising from the Schwann cells of the peripheral nerve sheath, with an estimated incidence of 0.3–0.4 per 100,000 in the general population; although the vast majority are sporadic, they may occur in the context of neurofibromatosis type 2 or schwannomatosis [1,2]. Retroperitoneal schwannomas are considerably rarer, accounting for 1–3% of all schwannomas and tend to grow slowly within the compliant retroperitoneal space, often reaching large dimensions before producing symptoms [3]. Clinical presentation is typically characterized by non-specific pain or, in cases of significant organ displacement, compression-related symptoms [3,4]. The absence of pathognomonic imaging features makes pre-operative diagnosis challenging, and accurate differentiation from other retroperitoneal masses, including neurofibroma, paraganglioma, liposarcoma, leiomyosarcoma, and lymphoma, requires integration of clinical, radiological, and histopathological findings [4,5]. Within this group, giant invasive spinal schwannomas represent a particularly challenging subtype. They are defined by involvement of two or more adjacent vertebral segments, an extraspinal component exceeding 2.5 cm, and vertebral body erosion [6]. Because of their slow growth, these lesions are frequently discovered incidentally as large masses, only becoming symptomatic when nerve compression or spinal involvement produces clinically relevant findings [7,8].

Surgical resection is the treatment of choice, as these tumors respond poorly to chemotherapy and radiotherapy [9]; however, their proximity to major abdominal vessels and spinal structures poses significant intraoperative challenges [4].

Vertebral body erosion is a particularly demanding finding when planning surgical resection, as the extent of bony involvement determines whether tumor removal will result in spinal instability requiring fixation. No formal guidelines exist on this matter, and the available evidence is limited to case reports and small series. A 25% vertebral body involvement threshold has been proposed as an indication for instrumentation, though this criterion does not account for the potential biological adaptation of bone to chronic mechanical loading [6,10].

An underexplored factor in this context is bone quality: experimental and clinical studies have shown that long-standing mechanical compression can activate biochemical pathways involved in bone matrix remodeling, leading to increased osteoblastic activity and cortical thickening of the eroded vertebral body as a compensatory response to chronic loading, potentially preserving structural integrity despite significant osseous involvement [11].

Hounsfield Unit (HU) measurements on CT have been used as a surrogate for bone mineral density, with consistent correlations to dual-energy X-ray absorptiometry (DEXA) and prognostic utility in the context of spinal surgery [12,13,14,15]. Elevated HU values reflect denser, more mineralized bone, while low values have been associated with an increased risk of implant failure, screw loosening, and adjacent segment fracture [13]. Because CT is routinely obtained in the pre-operative workup of these patients, HU measurement represents an immediately available and easily reproducible parameter that does not require additional imaging [16].

We report the case of a 56-year-old man with a giant retroperitoneal lumbar schwannoma causing 36.6% erosion of the L5 vertebral body, managed with complete tumor resection via an anterior laparotomic approach without spinal fixation. Post-hoc Hounsfield Unit measurements on pre-operative CT revealed compensatory bone sclerosis at the eroded vertebral level, highlighting the potential role of HU assessment in guiding surgical decision-making regarding spinal instrumentation.

## 2. Case Presentation

A 56-year-old man with a history of multilevel disc degeneration and benign prostatic hyperplasia presented with bilateral gluteal pain and urinary urgency. In 2016, he underwent L4–L5 microdiscectomy, at which time pre-operative imaging had incidentally revealed a left paravertebral mass measuring 55 × 40 × 35 mm anterior to the L5 vertebral body, consistent with schwannoma. Given the absence of symptoms attributable to the mass at that time, conservative management with radiological surveillance was adopted.

In May 2024, he developed bilateral gluteal pain—described as shock-like episodes occurring in the recumbent position—along with pain radiating to the medial aspect of both thighs, and urinary urgency. Neurological examination revealed intact motor and sensory function, symmetrically reduced deep tendon reflexes, and no gait instability, consistent with the slow-growing nature of the tumor and its origin from sensory rootlets of the left L5 nerve root.

Abdominal ultrasound followed by CT revealed a giant retroperitoneal mass measuring 97 × 67 mm with progressive erosion of the L5 vertebral body and anterior displacement of the left common iliac artery.

### 2.1. Diagnostic Workup

The case was reviewed by our multidisciplinary sarcoma team, which confirmed the indication for surgical biopsy. CT-guided core needle biopsy performed in October 2024 established the diagnosis of schwannoma: mitotic count below 1 per 10 high-power fields, Ki-67 proliferative index below 5%, no necrosis, and immunohistochemistry positive for S100 protein and SOX10, negative for CD34 and EMA, consistent with a benign peripheral nerve sheath tumor according to the WHO 2020 classification [17].

^18^F-FDG PET/CT was subsequently obtained on oncological advice and demonstrated marked and heterogeneous radiotracer uptake within the lesion (SUVmax 8.5), involving the left hemi-soma of L5 and extending across the midline. Importantly, no pathological lymph node uptake was identified in the iliac-obturator or lumbo-aortic regions, and no distant sites of increased glycolytic activity were detected.

Pre-operative planning included MRI and angio-CT to characterize the relationship of the tumor with the dural sac, nerve roots, and major vessels. MRI confirmed a mass measuring 82 × 85 × 63 mm with extensive scalloping and erosion of the left hemi-soma of L5, involvement of the postero-lateral wall of the vertebral body, and encroachment of the left L5–S1 neural foramen. The left common iliac artery, its branches, and the left ureter were displaced anteriorly; the left psoas muscle was displaced antero-laterally (Figure 1). Quantitative assessment of vertebral body involvement on CT demonstrated the erosion of the L5 soma, while contrast-enhanced sequences confirmed anterior displacement of the left iliac vessels (Figure 2).

Volumetric segmentation of L3, L4 and L5 vertebral bodies was performed using ITK-SNAP software (v3.8.0). Furthermore, Hounsfield unit (HU) analysis was conducted to determine the mean density of the remaining L5 vertebral body; computing of the vertebral body of L3, L4 and the remaining part of L5 was performed by thresholding HU values within three dedicated ROIs from the pre-operative CT scan and subsequent manual segmentation; Table 1 summarizes the computed volumes and mean HU relative to each vertebral body.

For each vertebral body, a ROI was manually computed in all three axes. To minimize observer variability, the semi-automatic active contour evolution algorithm was utilized. The initial 3D region-growing mask was constrained by strict Hounsfield Unit (HU) thresholding (150 HU lower limit with no upper limit) to isolate trabecular and cortical bone from surrounding soft tissues. Following the algorithmic segmentation, manual refinement was performed to ensure precise anatomical boundaries, specifically excising the posterior elements at the base of the pedicles to isolate the vertebral body. Finally, the mean voxel intensity, representing the mean radiodensity (HU) of the bony tissue, was extracted for each 3D vertebral model using the software’s native volumetric and statistical calculation tools for comparative analysis. The original volume of the L5 vertebral body was estimated at 24,328 mm^3^ by doubling the volume of the right hemisoma, which was unaffected by the tumor. Based on these calculations, a 36.6% erosion of the L5 vertebral body was identified.

Figure 3 shows the ITK-Snap working interface with a 3d-rendition of the computed vertebral bodies.

Furthermore, Hounsfield unit (HU) analysis was conducted to determine the mean density of the remaining L5 vertebral body; computing of the vertebral body of L3, L4 and the remaining part of L5 was performed by thresholding HU values within three dedicated ROIs from the preoperative CT scan and subsequent manual segmentation.

Table 1 summarizes the computed volumes and mean HU relative to each vertebral body.

While mean density of the healthy vertebral bodies of L3 and L4 were similar, mean HU of the remaining part of L5 was significantly higher, with an overall HU ratio of 1545.

### 2.2. Surgical Approach

The operation was performed in April 2025 under general anesthesia with continuous intraoperative neurophysiological monitoring, in a combined neurosurgical and general surgery setting. With the patient in the supine position, a supraumbilical-to-suprapubic midline laparotomy was performed. Upon entry into the peritoneal cavity, the tumor was confirmed as an approximately 8 cm mass occupying the left hemipelvis, lying anterior to the L5 vertebral body and sacrum. The left paracolic gutter was incised to access the left retroperitoneal space, and the left ureter was identified and preserved throughout.

The lesion was found to develop posterolateral to the left iliac vessels, with dense adhesions to the left internal and external iliac arteries and to the left common and external iliac veins, all of which were markedly displaced medially. Progressive and meticulous dissection was performed to separate the tumor from the iliac vessels. The surgical field was then handed to the neurosurgical team.

The tumor capsule was cauterized and incised. Progressive piecemeal debulking was performed to reduce tumor bulk and facilitate dissection within the presacral space. The left L5 and S1 nerve roots were identified and preserved. The tumor was found to originate from posterior sensory rootlets of the left L5 nerve root; these were individually mapped by direct intraoperative electrophysiological stimulation and, in the absence of any evoked response, coagulated and sectioned. Dissection was completed by excising the tumor component embedded within the L5 vertebral body cavity, achieving macroscopically complete removal. An abdominal drain was placed. No spinal fixation was performed.

Final histological examination confirmed a giant schwannoma of the peripheral nerve sheath (mitotic count <1/10 HPF; no necrosis), consistent with a benign lesion.

### 2.3. Postoperative Course and Follow-Up

The postoperative course was uneventful. Early mobilization was achieved on the first postoperative day with the use of an abdominal brace; pre-operative pain resolved completely, and no new neurological deficits were observed. The abdominal drain was removed once drainage had become minimal, and the patient was discharged on postoperative day seven in stable clinical condition.

Post-operative contrast-enhanced CT confirmed macroscopically complete tumor resection. (Figure 4).

At one-month follow-up, the patient reported complete resolution of gluteal and medial thigh pain and demonstrated no motor or sensory deficits on neurological examination. Bladder function had improved relative to the pre-operative status. At the successive follow-up appointments at 6 and 12 months, the patient was neurologically stable and did not report any new symptom. Long-term radiological surveillance is planned to monitor both oncological status and spinal alignment.

## 3. Discussion

Giant lumbar retroperitoneal schwannomas with vertebral body erosion are exceedingly rare; the published literature on this specific combination is largely confined to isolated case reports and small series [7,8,10]. Their slow growth within the compliant retroperitoneal space allows them to attain considerable size before producing symptoms, which accounts for the frequent delay in diagnosis and the advanced tumor dimensions at presentation. In this patient, the lesion had been known and monitored for eight years before symptoms and radiological progression prompted the indication for surgery, a timeline consistent with the natural history reported in similar cases [8,10].

Pre-operative diagnosis of retroperitoneal schwannomas remains challenging due to the absence of pathognomonic imaging features and the broad differential diagnosis, which includes neurofibroma, paraganglioma, liposarcoma, leiomyosarcoma, lymphoma, and retroperitoneal metastases [5]. In our case, CT-guided biopsy established the diagnosis, with immunohistochemical positivity for S100 and SOX10, a low Ki-67 index, CD34 negativity, and EMA negativity confirming a benign peripheral nerve sheath tumor and excluding malignant transformation [5].

18F-FDG PET/CT demonstrated marked radiotracer uptake (SUVmax 8.5); however, high FDG avidity is well documented in benign schwannomas and cannot reliably indicate malignancy when isolated [18]. The absence of distant uptake and lymph node involvement supported the decision to proceed with surgery.

The surgical approach in giant retroperitoneal schwannomas is dictated by tumor location, size, and relationship to adjacent structures [19]. Posteriorly confined lesions can be addressed via laminectomy, and lateral dumbbell tumors via foraminotomy or the Wiltse approach. Tumors with significant anterior retroperitoneal extension, however, require either a lateral retroperitoneal or an anterior laparotomic approach [19,20]. For schwannomas with significant sacral extension, combined trans- and extrasacral approaches have been described as an alternative strategy to achieve complete resection while preserving sacropelvic biomechanics [21].

In the present case, the marked anterior displacement of the left common iliac artery documented on pre-operative angio-CT guided the choice of a midline laparotomic approach. The anterior laparotomic approach offers wide operative exposure and direct visual control of the major iliac vessels, facilitating prompt management of vascular injury if needed, although its main drawbacks include the need for transperitoneal access and the associated risk of bowel-related complications. Conversely, while a lateral retroperitoneal approach would have avoided peritoneal entry and preserved abdominal wall integrity, the limited working space and reduced vascular visualization inherent to this route were considered unacceptable given the degree of iliac vessel displacement in this case [22,23]. The anterior approach was therefore selected as the safer option for this specific anatomical configuration.

The management of vertebral body erosion in giant schwannomas remains one of the most challenging intraoperative decisions, with no formal guidelines to direct surgeons. A 25% vertebral body involvement threshold has been proposed as an indication for spinal instrumentation, and this criterion has been referenced in subsequent case reports [6,7,8,10]. In the present case, erosion of 36.6% of the L5 soma exceeded this threshold. Nevertheless, instrumentation was not performed: despite the degree of osseous involvement, radiological instability was excluded on the basis of preserved intervertebral disc height, intact posterior ligamentous complex, and markedly elevated HU values at the eroded vertebral body, indicative of compensatory sclerosis sufficient to maintain load-bearing capacity. This decision was supported by the convergence of multiple favourable factors beyond HU values alone: the absence of axial mechanical pain or clinical signs of instability, the indolent tumour growth over eight years allowing adaptive tissue remodelling, the histological confirmation of a benign schwannoma, and a surgical strategy designed to preserve load-bearing bone, ligamentous, and muscular structures.

It is important to note that the 25% threshold derives from a limited series of ten patients and has not been validated prospectively [6]. Other published series have demonstrated stable outcomes after resection without instrumentation even in the context of substantial vertebral body erosion, provided that disc, endplate, and ligamentous structures remain intact [20,24]. Ozdemir et al. reported that none of six patients with giant erosive spinal schwannomas required instrumentation, identifying disc capsule and ligamentous integrity as the key determinants of post-operative stability [20]. Similarly, Yu et al. observed that instrumentation was required in a minority of cases in a series of 14 giant invasive spinal schwannomas, and that its necessity correlated more with disruption of the posterior elements than with the degree of vertebral body erosion alone [24]. When stabilization is ultimately required, modified open techniques designed to minimize soft tissue disruption while ensuring construct stability have been described, offering a less invasive alternative even in oncological settings requiring direct sacral access [25].

The long-term biomechanical consequences of leaving a vertebral body defect without instrumentation deserve consideration. Anterior column defects alter load distribution across the lumbar spine, and biomechanical studies have demonstrated that the degree of vertebral body involvement directly correlates with reduced construct rigidity and increased risk of progressive deformity over time [26]. In the present case, the compensatory sclerosis documented at L5 and the integrity of the posterior ligamentous complex provide reassurance regarding short-term stability; however, the durability of this equilibrium under physiological loading, ageing-related bone density changes, or accidental trauma remains unknown. Long-term radiological surveillance is therefore recommended, including standing full-spine imaging to detect any progressive sagittal or coronal malalignment. Non-invasive assessment tools such as three-dimensional posturography have shown promise in monitoring dynamic lumbosacral alignment parameters in the postoperative setting and may represent a valuable complement to conventional imaging in the longitudinal follow-up of these patients [27].

The present case adds an important dimension to this discussion through the use of HU measurements as an objective, pre-operative indicator of bone quality. HU values obtained from CT have been extensively studied as a surrogate for bone mineral density, demonstrating consistent correlation with DEXA T-scores and significant prognostic utility for outcomes in spinal instrumentation, including pedicle screw fixation failure, cage subsidence, and adjacent segment fracture [12,13,14,15,28,29,30,31]. In this patient, HU measurement in the L5 vertebral body yielded a mean value of 480 HU, compared with 317 HU at the adjacent L4 vertebra. This approximately 1.5-fold increase in density at the eroded level is consistent with reactive sclerosis—a recognized biological response to chronic mechanical loading, in which osteoblastic pathways are activated, leading to increased bone matrix deposition and cortical thickening [11].

A systematic review of HU values in lumbar vertebrae suggests a physiological range of approximately 100–200 HU for non-osteoporotic bone in adults, with the threshold for osteoporosis typically placed around 110–135 HU at L1 [16]. The absolute L5 HU value of 480 HU in our patient was therefore not only markedly elevated relative to the reference vertebra, but substantially above the range of physiological normality, indicative of significant sclerotic change. It should be noted that most studies establishing HU reference values have focused on L1–L4, and L5 values cannot be directly extrapolated; nonetheless, the magnitude of the inter-level difference in this case is striking and strongly suggests a pathological increase in bone density secondary to the chronic compressive effect of the schwannoma.

Although this interpretation is based on a single retrospective observation and cannot be generalized beyond this case, it supports the hypothesis that long-term chronic compression by a slow-growing tumor can induce compensatory bone remodeling sufficient to preserve vertebral structural integrity. If validated prospectively, pre-operative inter-level HU ratio assessment could represent a practical and immediately available adjunct to guide instrumentation decisions. This would be particularly valuable in cases where erosion exceeds conventional thresholds, but no clinical or radiological instability is present. HU measurements require no additional imaging and can be performed on the CT scans routinely obtained for pre-operative planning. Their integration into the pre-operative assessment of these patients appears clinically justified, though the evidence base currently rests on individual observations and requires corroboration through larger series.

Intraoperative neurophysiological monitoring is widely employed in spinal tumor surgery to reduce the risk of new neurological deficits and to guide the extent of resection [32]. In schwannomas, the nerve root of tumor origin can often be sacrificed without producing clinically significant neurological deficit, as the slow growth of the tumor allows adjacent nerve fibers to progressively compensate for the function of the affected root [33]. Safaee et al., in a series of 221 spinal nerve sheath tumors, reported that root sacrifice did not significantly worsen neurological outcomes in patients where pre-operative deficits were already present or where intraoperative stimulation demonstrated absent electrophysiological responses [33].

Although preoperative electromyography and nerve conduction studies were not performed, the clinical presentation and anatomical compression documented on CT and MRI provided sufficient grounds for surgical decision-making. Intraoperatively, the left L5 root was identified as the root of origin and found to produce no electrophysiological response on intraoperative stimulation; it was therefore sectioned to achieve complete tumor excision. The patient had no new motor or sensory deficits postoperatively. This outcome reinforces the value of intraoperative electrophysiological mapping as a tool not only for neural protection but also for confirming the functional redundancy of the affected root before committing to sacrifice.

## 4. Conclusions

Giant retroperitoneal schwannomas with vertebral body erosion exceeding the conventionally proposed 25% instrumentation threshold do not invariably require spinal fixation. In the present case, the absence of clinical and radiological signs of instability, preserved disc and ligamentous integrity, and markedly elevated HU values at the eroded vertebral body collectively supported a non-instrumented surgical strategy, with sustained clinical and radiological stability confirmed at one-year follow-up.

Hounsfield Unit measurements demonstrated a 1.5-fold increase in bone density at the eroded L5 level compared with the adjacent L4 vertebra, consistent with compensatory sclerosis induced by chronic mechanical compression. These findings support the hypothesis that long-standing tumor-related loading may activate bone remodeling pathways sufficient to preserve structural integrity despite substantial osseous involvement, though this interpretation is derived from a single retrospective observation.

Pre-operative HU assessment on CT represents a readily available, reproducible, and zero-cost tool that may complement anatomical erosion criteria in surgical decision-making. Prospective multicenter studies are needed to validate its role as a reliable guide to instrumentation in giant schwannomas with vertebral body involvement.

## Figures and Tables

**Figure 1:**
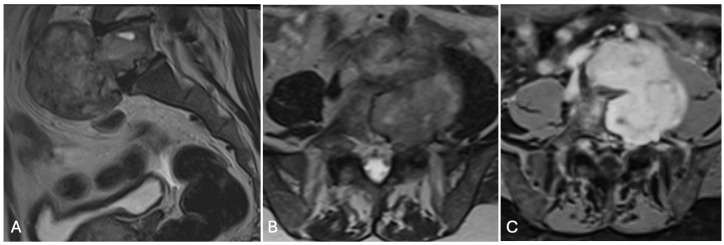
Pre-operative MRI of the lumbar spine. (**A**) Sagittal T2-weighted image demonstrating a large heterogeneous paravertebral mass with extensive scalloping of the L5 vertebral body. (**B**) Axial T2-weighted image showing the left paramedian retroperitoneal lesion with erosion of the left hemi-soma of L5, encroachment of the left L5–S1 neural foramen, and anteromedial displacement of the left iliac vessels and psoas muscle. (**C**) Axial T1-weighted contrast-enhanced image demonstrating intense and homogeneous enhancement of the lesion.

**Figure 2:**
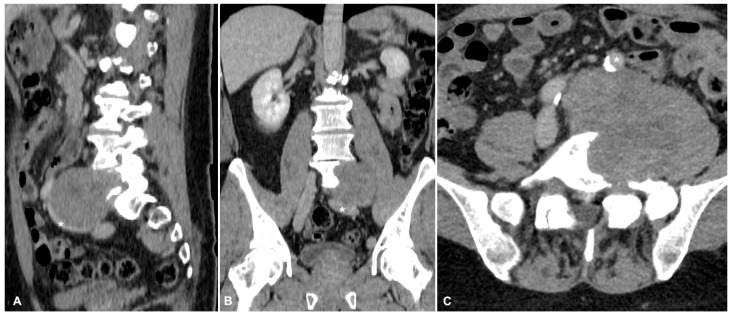
Pre-operative angio-CT. (**A**) Sagittal reconstruction demonstrating the extent of the erosion of the left hemi-soma of L5. (**B**) Coronal reconstruction demonstrating erosion of the left hemi-soma of L5. (**C**) Axial section illustrating osseous erosion of the left hemi-soma of L5 and encroachment of the left L5–S1 neural foramen. (*) indicating anterior and medial displacement of the left common iliac artery and its branches.

**Figure 3:**
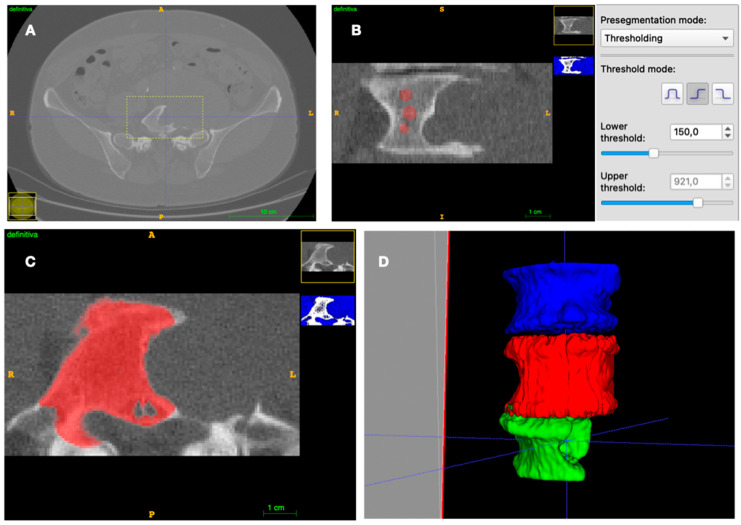
ITK-Snap working interface with a 3d-rendition of the computed vertebral bodies. (**A**) axial view of the ROI-identification process; the dashed yellow box delineates the region of interest selected for segmentation of the L5 vertebral body; (**B**) manual thresholding of HU values within the L5 ROI; (**C**) subsequent automatic algorithmic segmentation; (**D**) 3D rendering of the segmented L3 (blue), L4 (red), and L5 (green) vertebrae after manual refinement.

**Figure 4:**
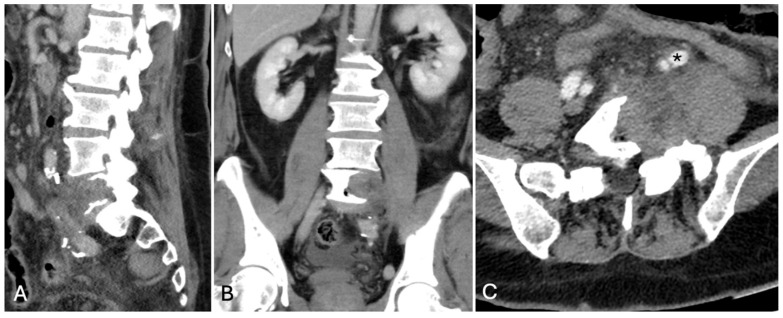
Post-operative angio-CT demonstrating complete resection of the retroperitoneal mass with restoration of the normal presacral and prevertebral space. (**A**) Sagittal reconstruction. (**B**) Coronal reconstruction. (**C**) Axial reconstruction. (*) Spontaneous repositioning of the left common iliac artery following relief of mass effect.

**Table 1:** Computed volumes and mean HU relative to each vertebral body.

Label Name	Number of Voxels	Volume (mm^3^)	HU Image Mean	HU Image Stdev
L4	60,782	44,309.7	317.282	192.444
L3	68,209	49,723.9	303.82	192.555
L5	33,373	24,328.7	480.141	205.24
Somatic Tumor	19,295	14,065.9	100.832	83.058

## Data Availability

The original contributions presented in this study are included in the article material. Further inquiries can be directed to the corresponding author.

## Abbreviations

The following abbreviations are used in this manuscript:

CT	Computed Tomography
DEXA	Dual-energy X-ray absorptiometry
FDG	Fluorodeoxyglucose
HPF	High-Power Field
HU	Hounsfield Unit
MRI	Magnetic Resonance Imaging
NF2	Neurofibromatosis type 2
PET	Positron Emission Tomography
ROI	Region of Interest
SUVmax	Maximum Standardized Uptake Value
